# Safe Placement of a Micro-Electromechanical Systems (MEMS), Levonorgestrel Releasing Contraceptive: Cadaver and Porcine Studies

**DOI:** 10.21203/rs.3.rs-11063684/v1

**Published:** 2026-10-23

**Authors:** Elizabeth Proos, Jeffrey Kalish, Brian J. Carmine, Nicolas Pacelli, Andrea Ries Thurman

## Abstract

In order to develop safe insertion criteria for future clinical studies of a levonorgestrel (LNG) releasing, wirelessly enabled micro-electromechanical system (MEMS)-based drug delivery device, we performed two pre-clinical studies in cadaver arms and pigs. Four female cadaver upper arms were dissected to assess the proposed insertion location, spatial orientation, and proximity to major muscle and neurovascular anatomy. Eight additional cadaver arms were dissected to assess insertion variables (incision depth, width, length, pocket creation). Twenty-four placebo MEMS devices were inserted sub-dermally into the backs of four miniature Yucatan pigs (6 devices per pig). Over a 2-to-4-month postoperative period, wound healing, device migration, and clinical observations were recorded. At removal, tissue capsule quality and tissue adhesions were evaluated. The target medial upper arm insertion site was 8–10 cm proximal to the medial epicondyle toward the axilla and 4–6 cm posterior to the brachial groove. Target insertion width (20 mm), depth (4 mm), and pocket length (51 mm) were defined. Porcine insertions were completed over 6–11 minutes with minimal bleeding. All sites healed well with no device migration over 2–4 months. After removal, tissue capsules were well formed and consistent with the expected normal healing response to a foreign body. These studies confirm the feasibility of safe insertion of the LNG MEMS device in the upper arm and a normal healing response to the device materials in live porcine tissue.

